# Efficient Bioconversion of Stevioside and Rebaudioside A to Glucosylated Steviol Glycosides Using an *Alkalihalobacillus oshimesis*-Derived Cyclodextrin Glucanotransferase

**DOI:** 10.3390/molecules28031245

**Published:** 2023-01-27

**Authors:** Ruiqin Zhang, Ruiqi Tang, Jiahua Bi, Shanshan Shen, Qin Wu, Qihe Chen, Yanjun Li

**Affiliations:** 1 Department of Food Science and Nutrition, Zhejiang University, Hangzhou 310058, China; 2 Key Laboratory of Food and Biological Engineering of Zhejiang Province, Research and Development Department, Hangzhou Wahaha Technology Co., Ltd., Hangzhou Wahaha Group Co., Ltd., Hangzhou 310018, China; 3 Key Laboratory of Bioprocess Engineering of Jiangxi Province, College of Life Sciences, Jiangxi Science and Technology Normal University, Nanchang 330013, China

**Keywords:** *Alkalihalobacillus oshimensis*, cyclodextrin glucanotransferase (CGTase), steviol glycosides, glucosylated steviol glycosides, transglycosylation

## Abstract

The enzymatic transglycosylation of steviol glycosides can improve the edulcorant quality of steviol glycosides. Cyclodextrin glucanotransferase (CGTase) is one of the most popular glucanotransferases applied in this reaction. Herein, the CGTase-producing strain *Alkalihalobacillus oshimensis* CGMCC 23164 was isolated from *Stevia* planting soil. Using mass spectrometry-based secretome profiling, a high-efficiency CGTase that converted steviol glycosides to glucosylated steviol glycosides was identified and termed CGTase-13. CGTase-13 demonstrated optimal transglycosylation activity with 10 g/L steviol glycoside and 50 g/L soluble starch as substrates at <40 °C. Under the above conditions, the conversion rate of stevioside and rebaudioside A, two main components of steviol glycosides, reached 86.1% and 90.8%, respectively. To the best of our knowledge, this is the highest conversion rate reported to date. Compared with Toruzyme^®^ 3.0 L, the commonly used commercial enzyme blends, glucosylated steviol glycosides produced using CGTase-13 exhibited weaker astringency and unpleasant taste, faster sweetness onset, and stronger sweetness intensity. Thus, CGTase provides a novel option for producing high-quality glucosylated steviol glycoside products and has great potential for industrial applications.

## 1. Introduction

Steviol glycosides are natural low-calorie sweeteners with high sweetness isolated from the leaves of *Stevia rebaudiana*. Steviol glycosides have attracted considerable attention from researchers and engineers in the food industry because they are natural, non-toxic, etc. [1,2,3]. However, the drawbacks are also obvious; its taste is slightly bitter and astringent, and the onset of sweetness is slower than that of sucrose [4,5,6].

Steviol glycosides are mixtures of diterpenoid glycosides, including stevioside (Stev), rebaudioside A (Reb A), rebaudioside D (Reb D), rebaudioside F (Reb F), rubusoside, and dulcoside A, and steviol aglycone is common in all the components [7,8,9,10]. The steviol aglycone has two active hydrogen molecules in the C13 hydroxyl and C19 carboxyl groups, which can perform intermolecular dehydration and glycogenesis reactions with the cyclic hemiacetal hydroxyl group of the sugar moiety to form various glycosidic compounds. Thus, steviol glycosides are formed on a common aglycone and steviol and differ only in the glycosidic constituents attached to C13 and/or C19 [11,12]. Studies have shown that the number of glucosyl groups attached to C13 and/or C19 considerably affects the taste profiles of steviol glycosides [11,12,13]. The two major sweet components of steviol glycosides are Stev and Reb A [13]. Stev was first discovered as the primary sweet component in *Stevia* leaves; however, it exhibited a strong bitter aftertaste [6,14,15,16]. It has been established that the sweetness and taste qualities of Stev can be substantially improved via glycosylation, for instance, Reb A (addition of one glucosyl group on the C13 of Stev), Reb D (addition of one glucosyl group on the C19 of Reb A), and Reb M (addition of one glucosyl group on the C19 of Reb D; [6]) taste better than Stev. Therefore, glycosylation is an approach for improving the taste profiles of steviol glycosides.

Cyclodextrin (CD) glucanotransferase (CGTase; EC 2.4.1.19) belongs to the α-amylase family 13 of glycoside hydrolases (GH13_2). With starch as the glycosyl donor, CGTase can transform steviol glycosides into glucosylated steviol glycosides [12,13,17,18,19,20,21,22]. The efficiency and components of the catalytic products of CGTase considerably affect the quality of glucosylated steviol glycosides. However, only a few CGTases are widely used in the market presently, leading to certain concerns, such as relatively fixed product components, low conversion rate, and high price, which substantially limit glucosylated steviol glycoside production.

Presently, Toruzyme^®^ 3.0 L from Novozymes is the most commonly used CGTase in the production of glucosylated steviol glycosides [14,23,24]. Therefore, this study was conducted with Toruzyme^®^ 3.0 L as a control. Herein, a CGTase-producing *Alkalihalobacillus oshimensis* strain was isolated from *Stevia* planting soil in Shandong Province, China. Using mass spectrometry (MS)-based secretome profiling, a CGTase was identified and termed CGTase-13. With soluble starch as the glycosyl donor, CGTase-13 transformed Stev and Reb A into glucosylated steviol glycosides with high efficiency. In addition, the glucosylated steviol glycoside products produced via CGTase-13 tasted better than those produced via Toruzyme^®^ 3.0 L. The findings of this study will provide novel perspectives to establish a high-efficiency transglycosylation system for the catalysis of steviol glycosides into glucosylated steviol glycoside products with better taste.

## 2. Results

### 2.1. Strain Isolation

**Figure 1:**
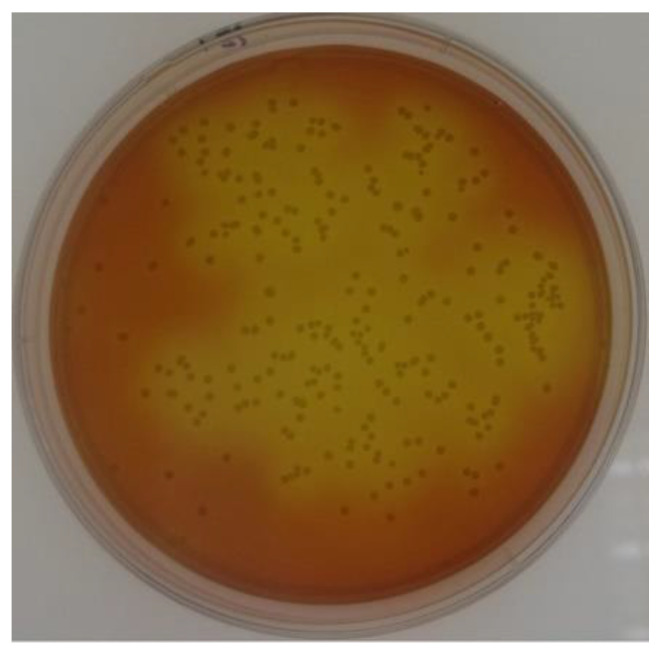
Colony morphology of *A. oshimensis*.

Among the 191 strains isolated from *Stevia* planting soil in the Shandong Province, five strains exhibiting CGTase activity were further screened and cultured using Horikoshi medium plate. CGTases are unique enzymes that catalyze the conversion of starch to CD, which absorbs the dye contained in the Horikoshi media and forms a yellow halo zone. After assessing the transglycosylation ability of steviol glycosides via high-performance liquid chromatography (HPLC) analysis, an efficient CGTase-producing strain (wahahaMZJ-1) was selected for further studies. Based on physiological and biochemical characterization, 16s rDNA, and gyrB results, the isolated wahahaMZJ-1 strain producing CGTase was identified as *A. oshimensis*. This strain was preserved at the China General Microbiological Culture Collection Center with CGMCC 23164. The colony morphology is shown in Figure 1.

### 2.2. Characterization of the CGTase Transglycosylation Product

**Figure 2:**
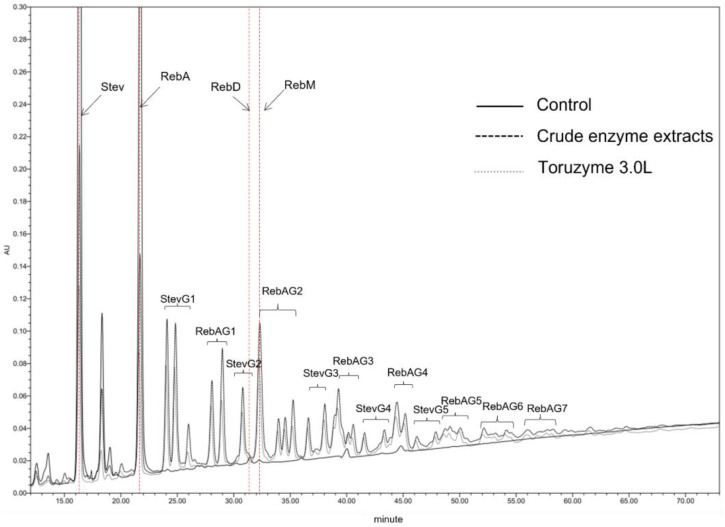
HPLC spectrum profiles of steviol glycosides and enzymatically modified steviol glycosides.

HPLC was used to detect the glucosylated steviol glycoside product (Figure 2). The extracellular enzyme of *A. oshimensis* wahahaMZJ-1 catalyzed various glucosylated steviol glycosides. Among them, the contents of the mono- and di-(α1-4)-glucosylated products were higher. Furthermore, compared with the products produced via Toruzyme^®^ 3.0 L, the products produced were different.

Because Reb D, Reb M, and their isomers are recognized as the components with better taste [6,25,26,27], a rough quantitative analysis was performed via ultrahigh-performance liquid chromatography-electrospray ionization–MS (UPLC–ESI–MS; Table 1).

As shown in Table 1, the peak areas of Reb D, Reb M, and their isomers catalyzed by the extracellular enzyme of *A. oshimensis* were significantly higher than those of the products catalyzed by Novozyme^®^ 3.0 L.

There is a phenomenon of “glucosyl group loss from mother ion (glucosylated derivatives were partially resolved)” in the process of MS (e.g., Reb M and Reb M isomer will become Reb D and Reb D isomer if one glucosyl group is lost). Despite this, the products catalyzed by *A. oshimensis* extracellular enzyme exhibited higher contents of Reb D, Reb M, and their isomers than the products catalyzed by Toruzyme^®^ 3.0 L. Therefore, it is speculated that a novel CGTase can be obtained from the extracellular enzyme for steviol glycoside modification, and the taste of the product is expected to be better than that produced via Toruzyme^®^ 3.0 L.

### 2.3. Heterologous Expression and Transglycosylation Activity Detection

The 12 possible CGTases were detected via liquid chromatography-tandem MS (LC–tandem MS/MS), as shown in Table S1.

The 12 CGTases were heterologously expressed and purified in *Escherichia coli*. Soluble starch (20 g/L) and steviol glycosides (20 g/L) dissolved in sterile water were incubated with 12 purified enzymes (20 mg/L) at 40 °C, and 220 rpm for 24 h and transglycosylation activity was detected. Among them, five CGTases exhibited relatively good transglycosylation activity (Table 2).

**Figure 3:**
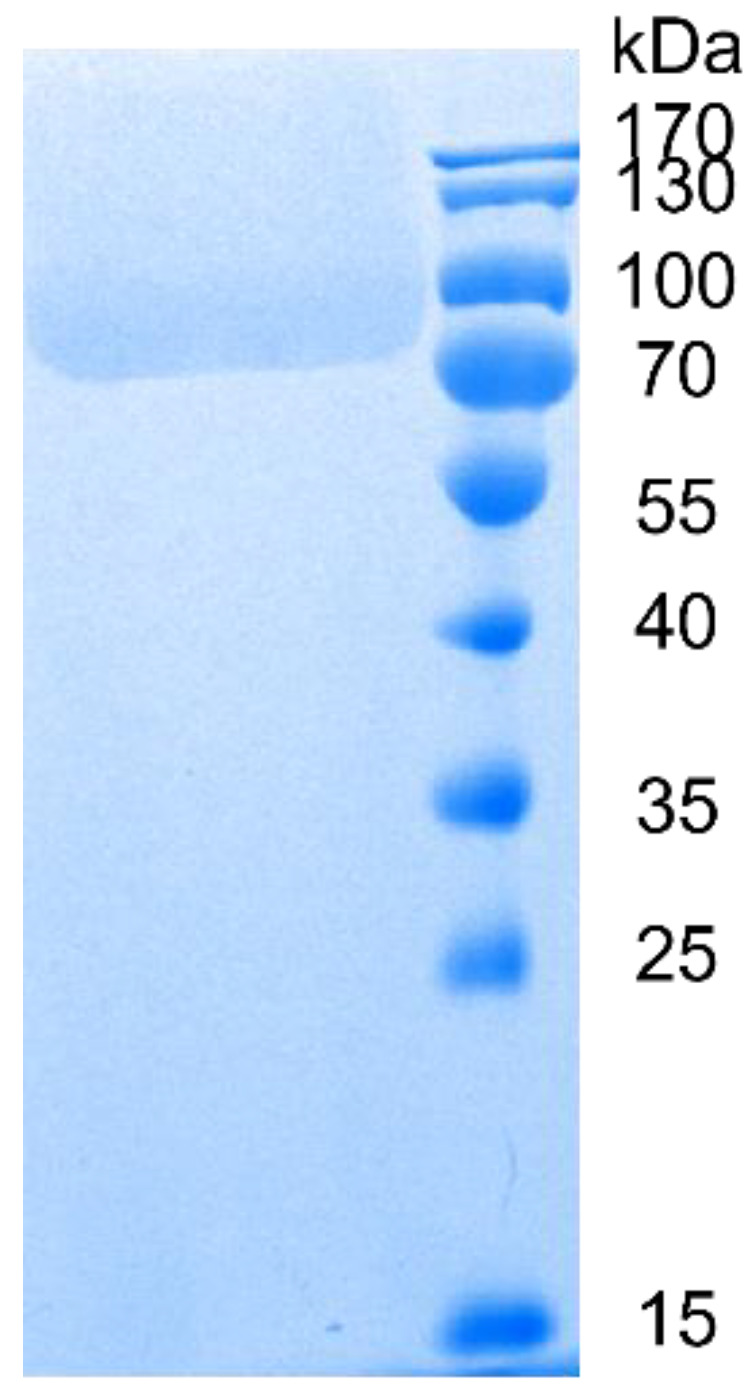
SDS–PAGE of CGTase-13.

Owing to its good performance, CGTase-13 was selected for further study, and it was expressed heterologously in *Pichia pastoris* GS115. The theoretical molecular weight of the CGTase-13 was 75.4 kDa. The recombinant CGTase-13 was analyzed by SDS–PAGE. The result is shown in Figure 3.

### 2.4. Determination of Product Specificity

Soluble starch solution (20 g/L) prepared in 50 mM Na_2_HPO_4_/NaH_2_PO_4_ (pH 6.0) was incubated with the enzyme sample (20 mg/L) at 40 °C and 220 rpm for 18 h. The reaction product was diluted 10 times, and the concentrations of α-, β-, and γ-CDs were determined. The results revealed that the concentration of α-, β-, and γ-CDs were 0.152 ± 0.007, 0.322 ± 0.024, and 0.056 ± 0.005 mg/mL, respectively; hence, CGTase-13 belongs to β-CGTase.

### 2.5. Effects of Temperature, pH, Incubation Time, and Substrate Concentration on the Conversion Rate

**Figure 4:**
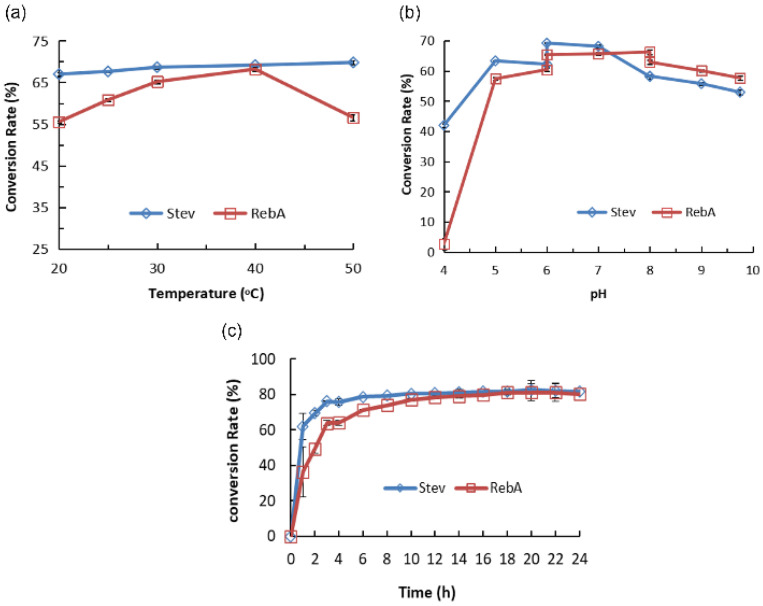
Effect of temperature (**a**), pH (**b**), and incubation time (**c**) on the conversion rate of Stev and Reb A to glucosylated steviol glycosides catalyzed by CGTase-13.

It is easy to see that the optimum temperature was 40 °C (Figure 4a). For Stev, in the pH range of pH 4–5, with the increase in pH, the conversion rate increased significantly. At pH 5–7, the conversion rate decreased slightly; however, the change was not obvious. When the pH exceeded 7, the conversion rate decreased significantly; thus, the optimal pH was 5 (Figure 4b). For Reb A, in the pH range of pH 4–5, with the increase in pH, the conversion rate increased significantly. At pH 5–6, the conversion rate increased slightly. At pH 6–8, the conversion rate remained almost unchanged. When the pH exceeded 8, the conversion rate exhibited a downward trend; thus, the optimal pH was 6–8 (Figure 4b). Therefore, according to the change range of the Stev and Reb A conversion rates, pH 6–7 was selected as the optimal pH.

For Stev, the conversion rate increased with the extension of reaction time within 0–6 h and did not change much after 6 h. For Reb A, the conversion rate increased rapidly with an increase in time from 0 to 10 h and slowly with an increase in time from 10–18 h. After 18 h, the conversion rate remained almost unchanged. Therefore, 18 h was selected as the optimum incubation time (Figure 4c).

**Figure 5:**
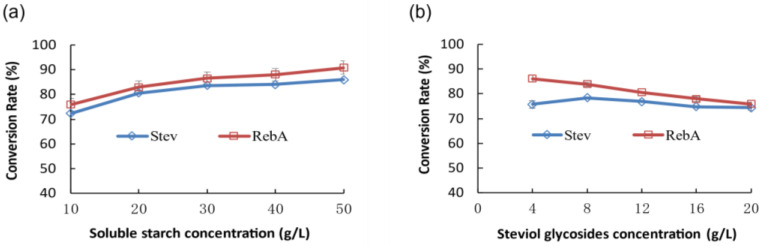
Effect of substrate concentration (soluble starch (**a**); steviol glycosides (**b**)) on the conversion rate of Stev and Reb A to glucosylated steviol glycosides catalyzed by CGTase-13.

Within the concentration range of 10–50 g/L, the conversion rates of Stev and Reb A gradually increase with an increase in soluble starch concentration. As the maximum solubility of the soluble starch used was ~50 g/L, it is impossible to continue to increase its concentration. Thus, 50 g/L was selected as the optimal concentration of soluble starch (Figure 5a).

Within the concentration range of 4–8 g/L, with an increase in steviol glycosides concentration, the Stev conversion rate increased slightly, whereas that of Reb A decreased slightly. When the steviol glycoside concentration exceeded 8 g/L, the conversion rates of Stev and Reb A exhibited a downward trend. Considering that the concentration of steviol glycosides used in actual production is generally ≥10 g/L, and the conversion rate of Stev and Reb A decreases by ≤5% when 8 g/L is compared with 12 g/L, 10 g/L was selected as the optimal concentration of steviol glycosides (Figure 5b).

Under optimum conditions, the conversion rates of Stev and Reb A reached 86.1% and 90.8%, respectively.

### 2.6. Sensory Profiles of Enzymatically Modified Steviol Glycosides

**Figure 6:**
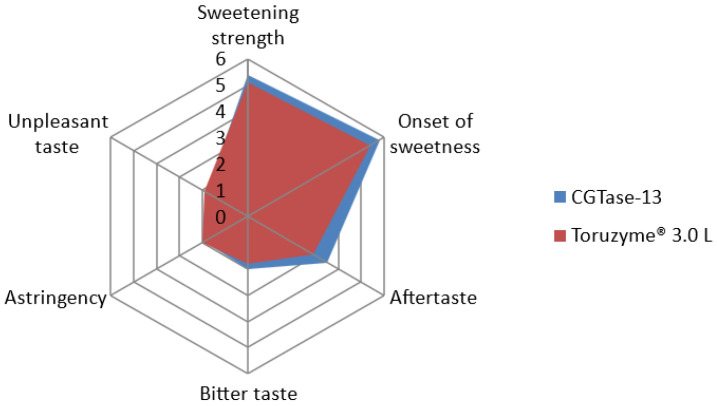
Spider-graph of sensory testing results (CGTase-13 and Toruzyme^®^ 3.0 L).

The results of the sensory analysis are shown in Figure 6. Compared with the products produced by Toruzyme^®^ 3.0 L, the glucosylated steviol glycoside product produced by CGTase-13 exhibited weaker astringency and unpleasant taste, faster sweetness onset, stronger sweetness intensity, and higher overall preference. The overall preference score of the CGTase-13 catalytic product was 71.3, and that of the Toruzyme^®^ 3.0 L product was 68.5. Thus, CGTase-13 improved the edulcorating quality of enzymatically modified steviol glycosides.

## 3. Discussion

Compared with steviol glycosides, an improvement in sweetness quality was observed in mono- and di-(α1-4)-glucosylated products. However, excessive glycosylation can affect the taste, such as decreasing sweetness and increasing bitterness [14,23]. Nevertheless, the number of transferred glucosyl groups of CGTase was random to some extent. Transglucosylation reactions yielded a mixture of mono- to multiple-(α1-4)-glucosylated products. Some studies have attempted to enhance the content of mono- and di-glucosylated products through control reaction conditions [14,23,28]. The UPLC–ESI–MS results revealed that the contents of mono- and di-glucosylated products catalyzed by the extracellular enzyme of A. oshimensis were significantly higher than those of the products catalyzed by Toruzyme^®^ 3.0 L. Therefore, it was speculated that there might be a novel efficient CGTase in the extracellular enzyme. Furthermore, there was a potential chance for improving the sensory profiles of steviol glycosides.

Numerous studies have employed CGTase alongside steviol glycosides as acceptor substrates to improve the gustatory characteristics of steviol glycosides [5,23]. The selection of the donor substrate is very important for the quality of products. CDs and starches provide the best transglucosylation yield [29]. Herein, CGTase-13 derived from A. oshimensis CGMCC 23164 was first identified, characterized, and applied for the transglycosylation of steviol glycosides using soluble starch as the donor substrate. In Table 3, a higher glucosylation rate was reported than in the literature. Considering the well-described mechanisms of transglycosylation activity and substrate specificity of CGTases, it is expectable that the transglucosylated products reported herein are based on the exclusive transfer of (α1-4)-glucose residues to the C-19-carboxyl and/or C-13 hydroxyl groups [10,14].

To investigate the impact of modification, a sensory analysis was conducted using Toruzyme^®^ 3.0 L as a control, consistent with the literature [14,23,24]. Compared with the product produced by Toruzyme^®^ 3.0 L, the glucosylated steviol glycoside product produced by CGTase-13 exhibited weaker astringency and unpleasant taste, faster sweetness onset, and stronger sweetness intensity. However, there remain some deficiencies in the aftertaste, such as a bitter taste, which warrant further improvement. This behavior resembles the results of Abelyan et al. [30], who, despite the effectiveness of transglycosylation, did not completely remove the bitterness and residual aftertaste in steviol glycosides modified with CGTases [12,30]. Thus, the overall preference for glucosylated steviol glycosides catalyzed by CGTase-13 was higher than that of the product catalyzed by Toruzyme^®^ 3.0 L. This indicates that CGTase-13 has obvious advantages compared with Toruzyme^®^ 3.0 L, thereby providing a novel option for producing glucosylated steviol glycosides, and has great potential for industrial applications.

Prior studies have established that coupling and disproportionation activities are directly associated with transglycosylation activity [32]. We selected CGTase-13, CGTase-15, and CGTase-7, which exhibit strong, medium, and weak transglycosylation abilities, respectively, and measured the coupling, disproportionation, hydrolysis, and cyclization activities of each enzyme. This established the degree of correlation between the strength of the transglycosylation ability of the enzyme and coupling, disproportionation, hydrolysis, and cyclization activities.

Table 4, Table 5, Table 6 and Table 7 present a tabulation of our results. The coupling activity of the CGTases in the descending order was CGTase-15 > CGTase-13 > CGTase-7. The disproportionation activity of the CGTases is in the descending order was CGTase-15 > CGTase-13 > CGTase-7. The hydrolysis activity of the CGTases in the descending order was CGTase-13 > CGTase-15 > CGTase-7 (all three CGTases exhibited weak hydrolytic activity). The cyclization activity of the CGTases in the descending order was CGTase-15 > CGTase-13 > CGTase-7. No correlation was found between the strength of the transglycosylation ability of the enzymes and any of the four reactions that were studied. We suspect that these four reactions jointly affect transglycosylation activity in a complex manner. It has already been established that the transglycosylation reaction does not include the substrates involved in the coupling and disproportionation activities; hence, our data may not reflect the true process of the transformation of steviol glycosides to glucosylated steviol glycosides.

## 4. Materials and Methods

### 4.1. Strain Isolation and Identification

Strain isolation and identification were adapted from Yu et al. [28], with minor modifications. The samples from the soil where *Stevia* was planted were collected from the Shandong Province (China) and diluted in a sterile dilution solution (0.9% saline). The aliquots were subsequently carefully positioned onto a Horikoshi medium plate and incubated for 3 days at either 28 °C or 37 °C. The isolates that produced CDs formed yellow halo zones. Once the isolates were definitively confirmed to secrete CGTases, the transformed products of steviol glycosides were analyzed via HPLC assays.

To identify each strain, each 16S rRNA gene sequence was amplified via polymerase chain reaction using the primer 27F/1492R (27F: 5′-AGAGTTTGATCCTGGCTCAG-3′ and 1492R: 5′-GGTTACCTTGTTACGACTT-3′). The amplicons obtained were sequenced, and the 16S rRNA gene sequences were aligned with the sequences from GenBank (http://www.ncbi.nlm.nih.gov/genbank/index.html, accessed on 10 February 2021.).

The 16S rRNA gene sequence of the strain was registered in GenBank nucleotide sequence databases with the accession number OP060998. The isolated strain was identified and deposited at the China General Microbiological Culture Collection Center with the strain number CGMCC 23164.

### 4.2. Protein Identification Using LC-Tandem MS (MS/MS)

Protein identification was performed at the APTBIO. Protein bands (70–100 kDa) were excised from the sodium dodecyl sulfate-polyacrylamide gel electrophoresis (SDS-PAGE) gel. A gel trypsin digestion method was performed to purify and prepare the gel piece (cut from an SDS-PAGE gel) before LC-MS/MS analysis.

The peptides were separated via EASY nLC and analyzed via MS using Q-Exactive (Thermo Scientific, Waltham, MA, USA). For LC analysis, separation was performed using a Thermo Scientific EASY column (2 cm × 100 μm, 5 μm C18) pre-column, followed by a Thermo Scientific EASY column (75 μm × 100 mm, 3 μm C18) at a flow rate of 300 nL/min. Full-scan MS spectra (m/z 300–1800) were acquired in the positive ion mode. MS files were searched against the Uniprot database (http://www.uniprot.org/, accessed on 7 May 2021.) using MaxQuant. Proteins were considered positively identified when they were identified with at least two tryptic peptides per protein. The false discovery rate was 1% for both peptides and proteins.

### 4.3. Heterologous Expression and Purification

The cDNA sequences of CGTases were codon-optimized according to *E. coli* codon bias and further synthesized and cloned in pET-28a(+) via GENEWIZ (Suzhou, China). The cDNA sequences of CGTase-13 were also codon-optimized according to *P. pastoris* codon bias and further synthesized and cloned in pPIC9K via GENEWIZ. The sequence of CGTase-13 optimized for *E. coli* was submitted to GenBank and assigned the temporary accession number OP095271. The sequence of CGTase-13 optimized for *P. pastoris* was submitted to GenBank and assigned the temporary accession number OP095272.

#### 4.3.1. Heterologous Expression and Purification in *E. coli*

The verified plasmids harboring the desired genes were transformed into *E. coli* BL21 (DE3) competent cells for expression. To induce expression, 0.1 mM IPTG was added to the media. After incubation, the cells were harvested via centrifugation (4500× *g* for 10 min). The harvested cells were suspended in sodium phosphate buffer (20 mM sodium phosphate, 200 mM NaCl, and 20 mM imidazole; pH 7.4). The cells were lysed on an ice bath via sonication, followed by centrifugation at 48,400× *g* for 30 min at 4 °C to remove insoluble cell debris. The recombinant proteins were purified via fast protein LC on an ÄKTA purifier equipped with 5 mL HisTrap affinity columns (GE Healthcare, Freiburg, Germany). The columns were equilibrated with binding buffer (20 mM sodium phosphate, 200 mM NaCl, and 20 mM imidazole; pH 7.4) at a flow rate of 2.5 mL/min. A 30 mL sample was loaded into the first column and extensively washed with binding buffer for five column volumes. The columns were then eluted with elution buffer (20 mM sodium phosphate, 200 mM NaCl, and 500 mM imidazole; pH 7.4). The purified enzymes were collected, and the elution buffer was replaced with protein buffer (50 mM sodium phosphate, pH 7).

#### 4.3.2. Heterologous Expression in *P. pastoris*

The verified plasmid was digested using the restriction enzyme SacI and transformed into *P. pastoris* GS115 via lithium chloride transformation. The transformants were selected, cultured in 25 mL BMGY media, and incubated at 28 °C until the density at 600 nm (OD_600_) reached a value of 2–6. The transformants were centrifuged at 4000× *g* for 5 min to collect the cells, which were then inoculated into 100–200 mL buffered methanol complex media (BMMY). Then, 1% (*v*/*v*) methanol was added at 24 h intervals to induce the expression of the heterologous protein. After 4 days of induction, the cell cultures were centrifuged at 10,000× *g* for 5 min at 4 °C, and the supernatant was collected.

### 4.4. Transformation of Steviol Glycosides with CGTase

Strain isolation and identification were adapted from Yu et al. [28], with minor modifications. Steviol glycosides (2 g) and soluble starch (2 g) were dissolved in 100 mL sterile water and divided into 2 mL centrifuge tubes (1 mL/tube). CGTase (20–21.2 mg/L) was added to the reaction solution, and the mixture was stored in a flask at 28–50 °C for 24 h to perform the reaction.

The conversion rate was calculated as follows [23,28]:

(1) $$Stev\;conversion\;rate\;\left(\%\right)=\frac{C_{o}-C_{t}}{C_{o}}\;\times 100\%$$

(2) $$Reb\;A\;conversion\;rate\;\left(\%\right)=\frac{C_{o}^{\prime }-C_{t}^{\prime }}{C_{o}^{\prime }}\times 100\%$$

where *C_o_*/*C_o_*′ is the initial *Stev*/*Reb A* concentration, and *C_t_*/*C_t_*′ is the detected *Stev*/*Reb A* concentration after the reaction.

The reaction mixture was monitored via HPLC (LC1200; Agilent Technologies, Inc., Santa Clara, CA, USA, and Waters Alliance e2695; Waters Corporation, Milford, MA, USA). The chromatographic column used for screening the strains was SB-C18 (4.6 × 250 mm; Agilent Technologies). Other transglycosylation reactions used Hypersil NH2 column (4.6 × 300 mm; Dalian Elite Analytical Instruments Co., Ltd., Dalian, China) for detection.

### 4.5. UPLC-ESI-MS

The UPLC-ESI-MS analysis was conducted using Waters Acquity UPLC-Xevo TQ MS. The UPLC conditions are presented in Table 8.

The MS parameters were polarity: ES-, full-scan mode, capillary: 3.5 kV, cone: 19 V, source temperature: 150 °C, desolvation temperature: 550 °C, cone gas flow: 60 L/h, and desolvation gas flow: 700 L/h.

### 4.6. Determination of Product Specificity

A 20 g/L soluble starch solution prepared in 50 mM Na_2_HPO_4_/NaH_2_PO_4_ (pH 6.0) was incubated with the enzyme sample (20 mg/L) at 40 °C and 220 rpm for 18 h. The reaction was quenched via incubation in a boiling water bath for 10 min. The reaction product was diluted 10 times, filtered through a 0.22-μm membrane, and analyzed using an evaporative light-scattering detector Alltech 3300 (1000254412; Waters Corporation).

The concentrations of α-, β-, and γ-CDs in the final sample were determined via UPLC using an Acquity BEH phenyl column (2.1 × 100 mm, 1.7 µm; Waters) and eluted with a methanol/water ratio of 1:99 at 0.3 mL/min.

### 4.7. Effects of Temperature, pH, Incubation Time, and Substrate Concentration on the Conversion Rate

Soluble starch (20 g/L) and steviol glycosides (20 g/L) dissolved in 50 mM Na_2_HPO_4_/NaH_2_PO_4_ (pH 6.0) were incubated with the enzyme sample (20 mg/L) at different temperatures (20 °C, 25 °C, 30 °C, 40 °C, and 50 °C) and 220 rpm for 24 h. The conversion rate was measured and calculated as described above.

The following buffers were used to evaluate the effect of pH on the conversion rate: 50 mM sodium acetate/acetic acid (pH 4–6), 50 mM Na_2_HPO_4_/NaH_2_PO_4_ (pH 6–8), and 50 mM Tris-HCl (pH 8–9.8). Soluble starch (20 g/L) and steviol glycosides (20 g/L) dissolved in a different pH buffer were incubated with the enzyme sample (20 mg/L) at 40 °C, 220 rpm for 24 h. The conversion rate was measured and calculated as described above.

Soluble starch (20 g/L) and steviol glycosides (20 g/L) dissolved in sterile water were incubated with the enzyme sample (20 mg/L) at 40 °C and 220 rpm for 0–24 h. The conversion rate was measured and calculated as described above.

Soluble starch (10, 20, 30, 40, and 50 g/L) and steviol glycosides (10 g/L) dissolved in sterile water were incubated with the enzyme sample (20 mg/L) at 40 °C and 220 rpm for 18 h. The conversion rate was measured and calculated as described above.

Soluble starch (20 g/L) and steviol glycosides (4, 8, 12, 16, and 20 g/L) dissolved in sterile water were incubated with the enzyme sample (20 mg/L) at 40 °C and 220 rpm for 18 h. The conversion rate was measured and calculated as described above.

### 4.8. Sweetness and Taste Evaluation via Sensory Methodologies

Sensory analysis was conducted by Zhucheng Haotian Pharmaceutical Co., Ltd., Zhucheng, Shandong. The experiments for sensory analysis were performed in a test room at a temperature of <30 °C. Eleven supertasters were recruited and requested not to eat or drink during the hour preceding testing, not to eat spicy foods during the previous day before each test, and not to use perfume on the day of testing. All stimuli were tested using a mouth sip-and-spit procedure. Each participant was provided with a cup of water. Participants were instructed to rinse their mouths between each sample and wait at least 2 min before continuing.

The evaluated samples were provided as solutions and included enzymatically modified steviol glycosides (product transformed by CGTase-13, 500 ppm) and enzymatically modified steviol glycosides (product transformed by Toruzyme^®^, 3.0 L, 500 ppm). Blind samples were provided to all the panelists for the sensory evaluation.

### 4.9. CGTase Activity Assays

The method to assess the disproportionation activity of the CGTases was adapted with minor modifications from Kong et al. [33]. The reaction conditions were optimized. The activity assay was completed under optimum conditions using 4-nitrophenyl-α-D-maltoheptaoside-4-6-O-ethylidene (EPS) and maltose as substrates. One unit of disproportionation activity was defined as the amount of CGTase required to convert 1 μM of EPS/min.

The method to assess the coupling activity of the CGTases was adapted with minor modifications from van der Veen et al. [34]. The reaction conditions were optimized. The activity assay was completed under optimal conditions using CD (α-, β-, γ-CD, respectively) and methyl α-D-glucopyranoside (M α-DG) as substrates. One unit of coupling activity was defined as the amount of CGTase required to couple 1 μM CD to M α-DG/min.

The method to assess the hydrolysis activity of the CGTases was adapted with minor modifications from Kong et al. [33]. The reaction conditions were optimized. The activity assay was completed under optimal conditions, and the hydrolysis activity was calculated from the difference between the quantity of reducing sugar produced by the hydrolysis reaction at 5 and 1 h. One unit of hydrolysis activity was defined as the quantity of CGTase required to produce 1 μM of maltose/min.

The method to assess the cyclization activity of the CGTases was adapted with minor modifications from previously described studies [35,36] under optimal conditions; γ-CD was not detected owing to producing an undetectable yield. One unit of cyclization activity was defined as the amount of CGTase required to produce 1 μM of α-CD or β-CD/min.

All activity data represents the mean value calculated from the results of three independent determinations.

## 5. Conclusions

The CGTase-producing strain *A. oshimensis* CGMCC 23164 was isolated from *Stevia* planting soil. Using MS-based secretome profiling, high-efficiency CGTase-13 was identified to convert steviol glycosides to glucosylated steviol glycosides. Under optimum conditions, the conversion rate of Stev and Reb A to glucosylated steviol glycosides catalyzed by CGTase-13 reached 86.1% and 90.8%, respectively. In addition, the sensory analysis revealed that the overall preference for glucosylated steviol glycosides catalyzed by CGTase-13 was higher than that for the product catalyzed by Toruzyme^®^ 3.0 L. Thus, modified steviol glycosides catalyzed by CGTase-13 might be more suitable sweetener substitutes for artificial and caloric sweeteners in food formulations.

## Figures and Tables

**Table 1:** Results of UPLC-ESI-MS detection of glucosylated steviol glycosides.

Sample	*m/z* 1289.5 (Reb M and Isomers)/Retention Time (min)/Peak Area							*m/z* 1127.5 (Reb D And Isomers)/Retention Time (Min)/Peak Area					
	1.6 min	2.1 min	2.4 min	2.8 min (Reb M)	3.4 min	3.8 min	4.8 min	1.7 min	2.4 min (Reb D)	2.9 min	3.3 min	3.9 min	4.5 min
Toruzyme^®^ 3.0 L	9980	23,341	27,473	81,265	44,730	6154	/	14,453	126,110	285,019	632,878	300,061	5298
*A. oshimensis*	12,472	2730	6664	298,013	46,509	69,650	/	11,103	39,968	49,779	785,395	348,797	34,404
Reb D 200 mg/L	/			/	/	/	/	/	75,051	/	/	/	/
Reb M 200 mg/L	/			219,969	/	/	/	/	/	/	/	/	/

**Table 2:** Conversion rate of Stev and Reb A.

Isolate No.	Stev Conversion Rate	Reb A Conversion Rate
13	80.4 ± 0.7%	79.2 ± 1.1%
8	76.9 ± 0.3%	77.3 ± 0.7%
15	77.4 ± 0.4%	63.1 ± 1.3%
14	69.7 ± 0.3%	69.8 ± 0.7%
7	65.6 ± 1.8%	52.9 ± 0.5%

**Table 3:** Summary of the enzymatic modification of steviol glycosides using CGTases.

Enzyme Source	Glycosyl Donor	Glycosyl Acceptor	pH	Temperature (°C)	Reaction Time	Reaction Type	Stev Conversion (%)	Reb A Conversion (%)	Reference
*Bacillus macerans* INMIA-BIO-4 m, *Bacillus circulans* INMIA-BIO-5 m, *Bacillus stearothermophilus* INMIA-B-4006, *Bacillus alcalophilus* INMIA-VA-4229, *Bacillus halophilus* INMIA-BIO-12N	Cyclodextrin	Stev	6.5–7.5	45, 32	20 h	Conventional	ND	ND	[30]
*Bacillus* sp. BL-12 β-CGTase	Maltodextrin	Stev	8.5	40	12 h	Conventional	76	ND	[19]
*Bacillus firmus* β-CGTase	β-cyclodextrin	Stev	1–11	10–80	1 min	Microwave reactor 80 W	70	ND	[5]
*Thermoanaerobacter* Toruzyme^®^ 3.0 L CGTase	Cornstarch	Stev	5–6	60	3 h	Conventional	77.11	ND	[23]
*T. aerobacter* Toruzyme^®^ 3.0 L CGTase, *Bacillus subtilis* α-amylase	Cyclodextrins and starches	Stev	-	-	5 h	Conventional	80	ND	[29]
*Paenibacillus* sp. CGMCC 5316 CGTase	Soluble starch	Stev	-	37	24 h	Conventional	85.6	ND	[28]
*T. aerobacter* Toruzyme^®^ 3.0 L CGTase	Gelatinized cornstarch	Stev	6–8	60	3 min	Microwave reactor 50 W	61.2	ND	[24]
*Trichoderma viridae* cellulase Onozuka R-10	Soluble starch, sucrose, lactose, glucose, β-cyclodextrin	*Stevia* leaf	4.6	50	10 h	Conventional	ND	ND	[31]
*Bacillus licheniformis* DSM 13 CGTase	Sucrose	Plant extract	3–9	14–45	16 h	Conventional	70–80	ND	[13]
*A. oshimensis* CGMCC 23164 CGTase-13	Soluble starch	Steviol glycosides	-	40	18 h	Conventional	86.1	90.8	This study

**Table 4:** Coupling activity of CGTases.

CGTase	Specific Enzyme Activity (with α-CD as the Glycosyl Donor) (U/mg)	Specific Enzyme Activity (with β-CD as the Glycosyl Donor) (U/mg)	Specific Enzyme Activity (with γ-CD as the Glycosyl Donor) (U/mg)
13	10.4 ± 0.6	24.9 ± 0.4	9.0 ± 0
15	149.4 ± 12.3	21.6 ± 0.7	30.1 ± 0.9
7	4.6 ± 0.1	9.1 ± 0.5	6.5 ± 0.2

**Table 5:** Disproportionation activity of CGTases.

CGTase	Specific Enzyme Activity (U/mg)
13	122.0 ± 1.7
15	248.0 ± 2.6
7	53.0 ± 1.1

**Table 6:** Hydrolysis activity of CGTases.

CGTase	Specific Enzyme Activity (U/mg)
13	0.5 ± 0.1
15	0.3 ± 0.0
7	0.0 ± 0.0

**Table 7:** Cyclization activity of CGTases.

CGTase	Specific Enzyme Activity (α-CD) (U/mg)	Specific Enzyme Activity (β-CD) (U/mg)
13	4.7 ± 0.6	17.3 ± 1.0
15	7.5 ± 0.4	12.8 ± 0.3
7	3.5 ± 0.4	12.8 ± 0.6

**Table 8:** UPLC conditions.

Time (min)	Water (%)	Acetonitrile (%)	Curve	Flow Rate (mL/min)
0	72	28		0.3
1.0	72	28	6	0.3
7.5	50	50	6	0.3
8.0	50	50	6	0.3
8.2	72	28	6	0.3
10	72	28	6	0.3

Injection volume: 5 μL. Column: Waters Acquity UPLC HSS T3 C18 (1.8 µm, 2.1 × 100 mm); temperature, 40 °C.

## Data Availability

Data are available on request from the authors.

## Supplementary Materials

The following supporting information can be downloaded at: https://www.mdpi.com/article/10.3390/molecules28031245/s1. Table S1. MS identification of CGTase.

## Sample Availability

Not available.
