# Supplementary material for: Efficient Bioconversion of Stevioside and Rebaudioside A to Glucosylated Steviol Glycosides Using an Alkalihalobacillus oshimesis-Derived Cyclodextrin Glucanotransferase
Source: Molecules. 2023 Jan 27;28(3):1245. doi: 10.3390/molecules28031245 (PMC9919944; doi:10.3390/molecules28031245)
Supplement: Supplementary file 1 [file molecules-28-01245-s001.zip › molecules-2098643-supplementary.pdf]

Table S1. MS identification of CGTase.

Isolate no.	Accession number	Description	Coverage	Unique peptides	MW (kDa)	Calc. pI
7	P31746	*Bacillus* sp. (cyclomaltodextrin glucanotransferase)	76.1	3	78.6	4.97
8	W7ZLX1	*Bacillus* sp. (cyclodextrin glycosyltransferase)	72.12	1	78.6	5.14
9	Q5U9W0	*Bacillus* sp. (cyclodextrin glycosyltransferase)	59.17	1	80.6	4.94
10	F1DPT1	*Bacillus* sp. (cyclodextrin glycosyltransferase)	70.42	2	80.6	5.00
11	A0A172MLH7	*Bacillus* sp. (cyclodextrin glycosyltransferase)	61.49	1	76.3	5.06
12	X0RDG2	*Bacillus* sp. (cyclodextrin glycosyltransferase)	17.05	3	78.5	4.49
13	W7YQ87	*Bacillus* sp. (cyclodextrin glycosyltransferase)	15.5	6	78.4	6.44
14	A0A1B3B4Y2	*Bacillus* sp. (cyclodextrin glycosyltransferase)	1.82	1	80.1	4.42
15	P31747	*Bacillus* sp. (cyclomaltodextrin glucanotransferase)	2.09	1	78	6.73
16	A0A0D0RA67	*Bacillus* sp. (cyclodextrin glycosyltransferase)	1.44	1	79.8	6.09
17	Q0JW31	*Alkalihalobacillus halodurans* (cyclomaltodextrin glucanotransferase)	1.45	1	75.8	6.27
18	A0A1S1FMF5	*Bacillus* sp. (cyclodextrin glycosyltransferase)	1.55	1	77.3	6.49

Accession number: protein number as found for the specific database. Description: protein name as assigned by the database. Coverage: percentage sequence coverage of the protein as detected by MS. Unique peptides: number of unique peptides detected for the specific protein. MW: molecular weight of the protein as given in the database. pI: pI of protein as given in the database.
